# Hydrogel research in peripheral nerve injury repair: a comprehensive multi-database bibliometric analysis (2015–2025)

**DOI:** 10.3389/fneur.2026.1763950

**Published:** 2026-04-29

**Authors:** Wenju Bai, Xiaoyuan Huang, Jian Liu, Hao Yue, Yaofeng Hu, Jinyong Li, Xu Zhang, Jichao Wang

**Affiliations:** 1 Department of Neurosurgery, People’s Hospital of Xinjiang Uygur Autonomous Region, Urumqi, Xinjiang, China; 2 Department of Orthopedics, People’s Hospital of Xinjiang Uygur Autonomous Region, Urumqi, Xinjiang, China; 3 Graduate School of Xinjiang Medical University, Urumqi, Xinjiang, China; 4 Xinjiang Second Medical College, Karamay, Xinjiang, China

**Keywords:** 3D bioprinting, angiogenesis, bibliometric analysis, conductive hydrogel, hydrogel, nerve guidance conduit, peripheral nerve injury, Schwann cells

## Abstract

**Background:**

Peripheral nerve injury (PNI) represents a significant clinical challenge with limited therapeutic efficacy. Hydrogel scaffolds, which mimic the natural neural extracellular matrix, have emerged as promising biomaterials for nerve regeneration. However, clinical translation is constrained by material stability issues and insufficient integration with host biological systems. This bibliometric analysis aims to map the evolving research landscape and identify emerging trends to guide future therapeutic development.

**Methods:**

A comprehensive literature search was conducted across Web of Science Core Collection (WoSCC), Scopus, PubMed from January 2015 to October 2025. Following deduplication and eligibility screening, 502 articles were included for bibliometric analysis. Publication trends, collaborative networks, and thematic evolution were analyzed using VOSviewer, CiteSpace, SCImago Graphica, and the R package bibliometrix.

**Results:**

The field demonstrated substantial growth over the decade, with publication volume increasing from 10 papers in 2015 to 80 in 2023. China dominated research output (55.0% of publications), though studies from the United States and Canada achieved higher average citation impact. Five major research clusters were identified, with significant focus on conductive hydrogels and Schwann cell-mediated regeneration. Thematic analysis revealed a paradigm shift from material characterization to mechanism-driven research, highlighting emerging interests in macrophage immunomodulation, angiogenesis, and nanofiber applications. Co-citation analysis indicated growing attention toward functionalized nerve guidance conduits and 3D/4D bioprinting technologies.

**Conclusion:**

Hydrogel-based PNI repair research has matured from basic scaffold development to integrated platforms combining electrical conductivity, advanced fabrication, and neural guidance engineering. Future priorities should emphasize stimuli-responsive “smart” hydrogel systems, pro-angiogenic strategies, and interdisciplinary collaboration to accelerate clinical translation.

## 1 Introduction

Peripheral nerve injury (PNI) continues to pose substantial challenges in clinical management, where high incidence rates lead to severe deterioration in patients’ quality of life. Common manifestations include chronic pain, sensory impairment, and loss of motor function, frequently arising from traumatic injury, surgical procedures, or underlying pathological conditions (1, 2). While autologous nerve grafts remain a commonly used intervention, limitations such as donor site complications, neuroma development, and unsatisfactory functional recovery persist (3–5).

**Figure 1:**
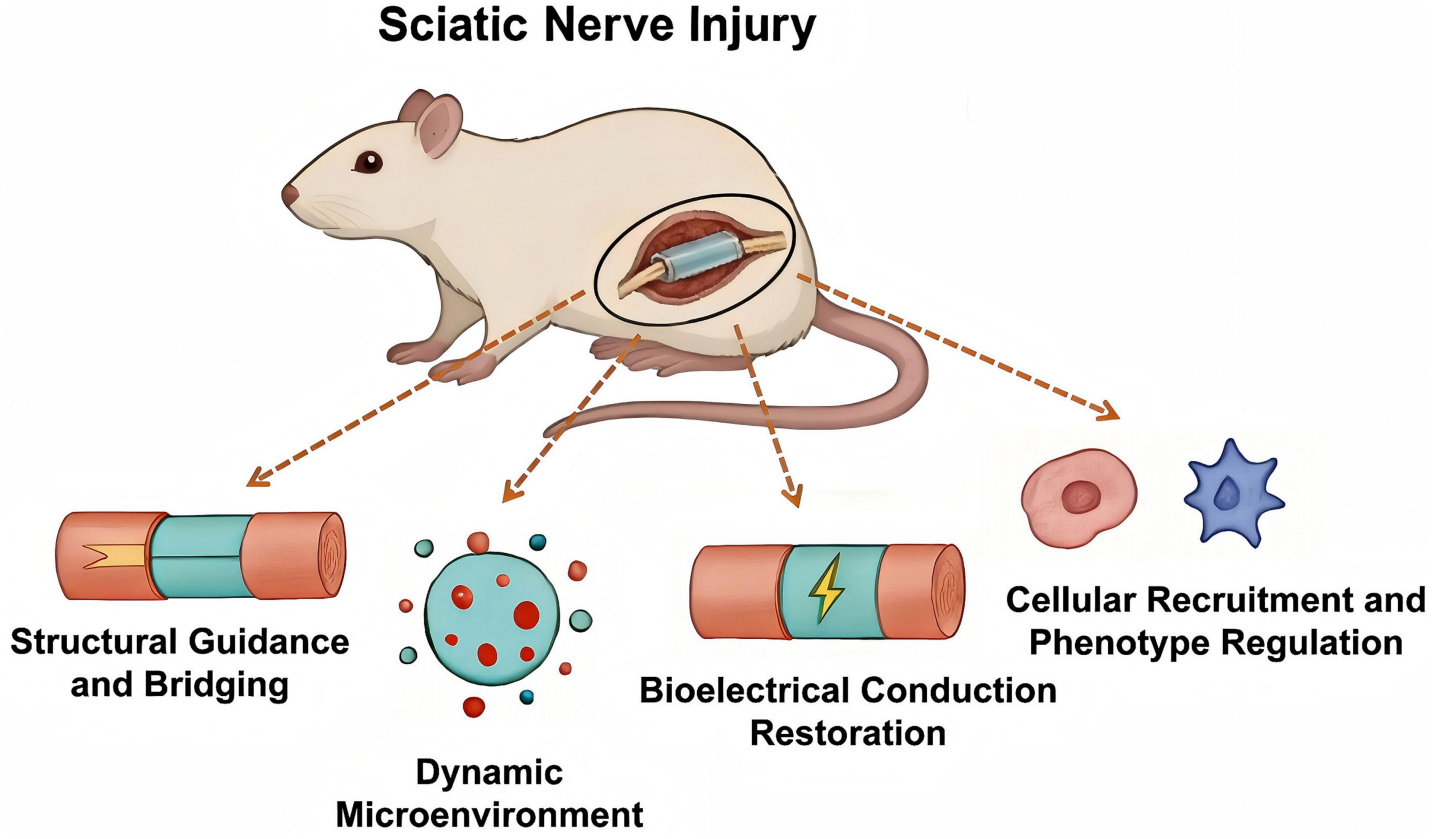
Key research axes in hydrogel-mediated rat sciatic nerve regeneration. The image was prepared using Bohrium (version 1.2.0).

Hydrogel-based biomaterials represent a promising direction in neural regeneration research, owing to their structural and functional similarity to native nervous tissue (6). These materials emulate the natural extracellular matrix (ECM) through three-dimensional cross-linked networks, offering a supportive environment for cellular adhesion, proliferation, and differentiation (7). Their high water content, efficient nutrient transport, and capacity for controlled delivery of bioactive agents collectively foster a conducive regenerative microenvironment (1). Furthermore, advances in material science allow fine-tuning of mechanical properties to better match those of injured neural tissues (8). In the context of PNI, hydrogels function not only as guidance conduits for axonal regrowth but also as carriers for neurotrophic factors and as barriers against excessive fibrotic scarring (9). Recent developments include hydrogels incorporating stem cells or Schwann cells, which promote regeneration through paracrine signaling and immunomodulatory mechanisms (10) (Figure 1).

Nevertheless, clinical translation faces several obstacles. Variability in hydrogel composition, unpredictable degradation profiles, and insufficient mechanical durability may hinder consistent therapeutic outcomes (11). Overcoming these limitations requires deeper investigation into the interactions between hydrogels and biological systems.

This review evaluates current applications of hydrogels in peripheral nerve repair, emphasizing recent advances, ongoing challenges, and potential future developments. Incorporating bibliometric insights from Web of Science Core Collection (WoSCC), Scopus, and PubMed (12), this work combines quantitative trends with qualitative interpretation to offer a holistic resource for researchers and clinicians engaged in developing biomaterial-based therapies for PNI.

## 2 Materials and methods

### 2.1 Data sources and search strategy

A systematic literature search was performed on 1 October 2025. The primary database was WoSCC including SCI-EXPANDED, CPCI-S, CCR-EXPANDED, and IC indices; supplementary searches were conducted in Scopus and PubMed. The search period spanned 1 January 2015 to 1 October 2025. Supplementary material 1 provides specific retrieval strategies.

### 2.2 Study selection and data preprocessing

We conducted a systematic literature search across three databases: WoSCC, Scopus, and PubMed. Equivalent search strings were adapted for each database. The initial search yielded 825 records (WoSCC *n* = 400, Scopus *n* = 290, PubMed *n* = 135).

**Figure 2:**
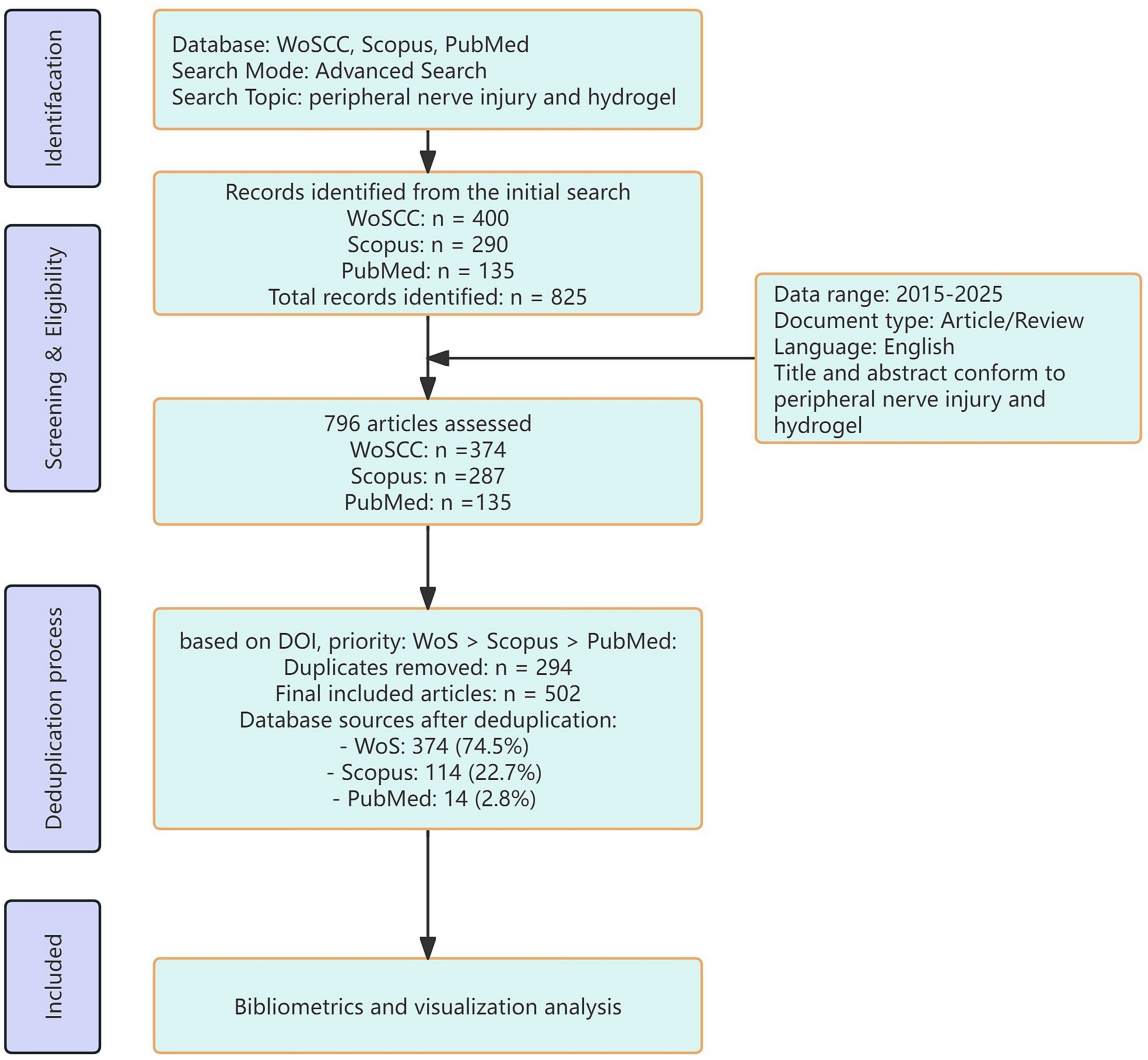
Flowchart of the article search.

After applying date range (2015–2025), document type (Article/Review) filters and language (English), 796 records remained (WoSCC *n* = 374, Scopus *n* = 287, PubMed *n* = 135) for deduplication. Records were excluded if they were conference abstracts, book chapters, editorials, or otherwise failed to meet inclusion criteria. Two reviewers independently screened titles and abstracts against predefined eligibility criteria: studies addressing hydrogels in peripheral nerve injury or repair; original articles or reviews; and publication within the study period. Disagreements were resolved by consensus or consultation with a third reviewer. Following deduplication, 502 unique articles were included for final analysis: WoSCC (*n* = 374, 74.5%), Scopus (*n* = 114, 22.7%), and PubMed (*n* = 14, 2.8%). We removed 274 duplicates identified through DOI matching, applying the following priority order for source attribution: WoSCC > Scopus > PubMed. Supplementary material 1 details the specific deduplication strategy. Figure 2 presents the complete PRISMA flow diagram.

### 2.3 Bibliometric analysis

The analytical process integrated several bibliometric tools: the bibliometrix package (v4.4.2) in R was used to evaluate publication and citation metrics, as well as collaboration networks among authors and institutions. CiteSpace (v6.4. R1) was applied to detect keyword bursts and generate co-occurrence and co-citation networks. VOSviewer (v1.6.20) enabled visualization of journal profiles and research clusters. SCImago Graphica (v1.0.48) mapped geographic distributions of international partnerships. Microsoft Excel was employed to compile descriptive statistics, produce time-series charts of publication and citation data, and build thematic trees. This combined toolset supported cross-validation of observations and yielded multi-level insight into author-, institution-, and country-level collaborations, plus evolving thematic directions.

### 2.4 Research ethics

Only published literature was examined in this bibliometric study. Because no human subjects or personally identifiable information were involved, institutional ethical review was not required.

## 3 Results

### 3.1 Publication volume and temporal trends

Bibliometric analysis, by examining scientific output and citation data, offers critical insights into a research field’s developmental trajectory, academic impact, and knowledge dissemination patterns.

**Figure 3:**
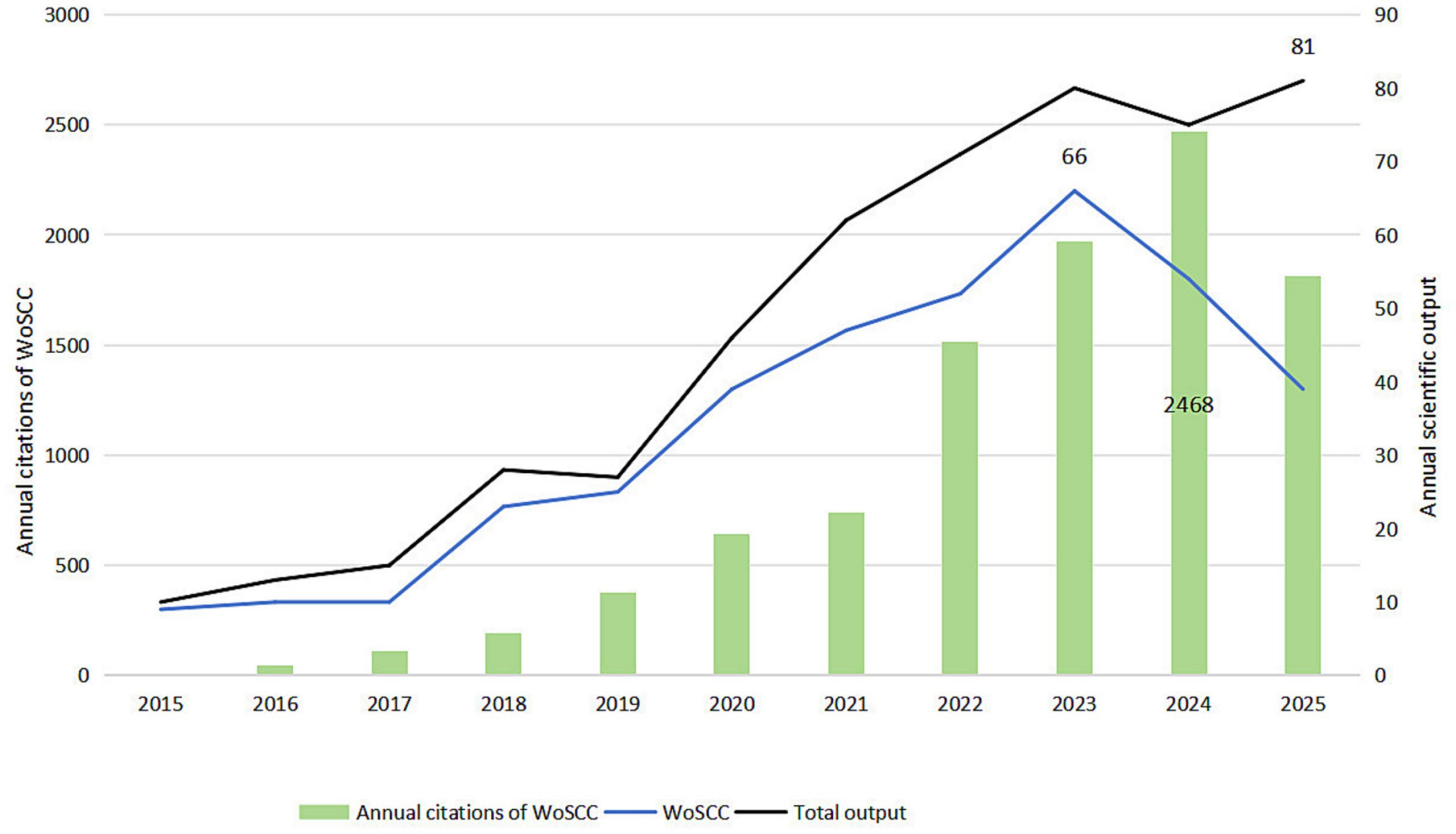
Annual scientific production and annual citations.

Figure 3 illustrates the annual publication and citation trends within “peripheral nerve injury + hydrogel” from 2015 to 2025. The blue line represents annual publications indexed in WoSCC; the black line shows the combined, deduplicated annual output from WoSCC, PubMed, and Scopus; and the bar graph depicts the annual citations of WoSCC-indexed publications.

Overall, the field has experienced substantial growth over the past decade. WoSCC publications steadily increased from 9 in 2015 to a peak of 66 in 2023. The combined annual output—reflecting a broader research landscape—also demonstrated a robust upward trend, climbing from 10 papers in 2015 to 80 in 2023. However, 2024 marked a slight reduction in output: WoSCC contributions decreased to 54, and the total combined output fell to 75. For 2025, WoSCC publications further decreased to 39, which is largely attributable to incomplete database indexing at the time of retrieval (October 2025). Of note, the total combined output for 2025 surprisingly rebounded to 81, surpassing the 2023 peak and indicating sustained research activity when considering multiple databases.

Research momentum visibly accelerated around 2020, reaching its zenith in 2023. This pattern not only signifies the field’s dynamic evolution but also underlines the necessity of multi-database retrieval for comprehensive trend analysis.

Concurrent with publication growth, the citation impact of WoSCC-indexed papers showed an even steeper ascent. Annual citations surged from a modest 8 in 2015, accelerating to 1,515 in 2022 (a 104% year-on-year increase from 2021). The highest annual citation count, 2,468, was recorded in 2024, representing an approximately 308-fold increase from 2015. Citations remained high in 2025 at 1,815. This robust citation profile suggests that high-impact studies published particularly during 2020–2021 garnered widespread recognition and influence in subsequent years.

### 3.2 Analysis of countries and regions

**Figure 4:**
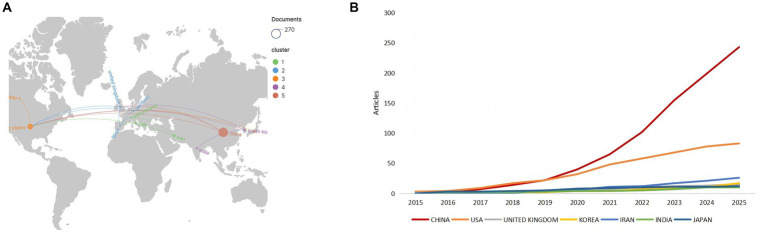
**(A)** Geographic collaboration network map of hydrogel and peripheral nerve injury research. Node size corresponds to publication volume; same color indicates the same cooperative cluster. **(B)** Cumulative line chart of research output achievements in major countries.

Country and regional analysis serves a fundamental role in bibliometric studies, offering critical insights into the global research landscape and revealing patterns of international collaboration within a field. It is important to note that all data presented in Table 1; Figures 4A,B are derived from a comprehensive, deduplicated dataset combining publications from WoSCC, PubMed, and Scopus.

**Table 1:** National research output in hydrogel-based peripheral nerve injury studies in WoSCC (2015–2025).

Rank	Country	Total citation	Average article citations	Documents	Rank	Country	Total citation	Average article citations	Documents
1	China	5,380	21.69	248	6	India	370	37.00	10
2	United States	1,398	15.53	90	7	United Kingdom	203	11.94	17
3	South Korea	553	29.11	19	8	Japan	202	16.83	12
4	Canada	518	57.56	9	9	Italy	152	15.20	10
5	Iran	443	16.41	27	10	Poland	132	18.86	7

As quantified in Table 1, which ranks countries by total aggregated citation count in the field of PNI and hydrogel research, China exhibits a dominant position. It leads unequivocally with 248 documents, constituting approximately 55.2% of the total output among the top ten countries, and has accumulated 5,380 total citations. While its average citations per article (21.69) are substantial, the United States, ranking second in both output (90 documents) and total citations (1,398), maintains a solid average citation rate of 15.53. Notably, Canada, despite a limited output of only 9 articles, achieved the highest average citations per paper (57.56), indicating outstanding research impact relative to its volume.

Using VOSviewer, we analyzed the top 12 countries by citation count, setting a minimum publication threshold of 7 articles per country. A co-authorship network was generated from the top countries by citation count, revealing five distinct clusters (Figure 4A). Cluster 1 (green) included Iran, Italy, and Switzerland. The blue cluster (Cluster 2) grouped Germany, Spain, and the United Kingdom. Cluster 3 (orange) comprised Canada, Japan, and the United States. India and South Korea formed the purple cluster (Cluster 4). Finally, China alone constituted the red cluster (Cluster 5).

Figure 4B, a cumulative line chart, provides a temporal perspective on the research output from the leading countries. It vividly illustrates China’s explosive growth in publication volume, particularly post-2020 (red line), which has surged far beyond all other nations. The United States (orange line) shows a steady, linear increase in cumulative publications, maintaining a consistent contribution over the decade. Other key countries, such as South Korea, Iran, India, and Japan, exhibit more modest but continuous growth trajectories.

### 3.3 Analysis of authors and institutions

Analysis of institutional research output pertaining to hydrogel applications for peripheral nerve repair was conducted using a comprehensive, de-duplicated dataset. This approach provided insights into publishing trends and collaborative structures among institutions.

**Figure 5:**
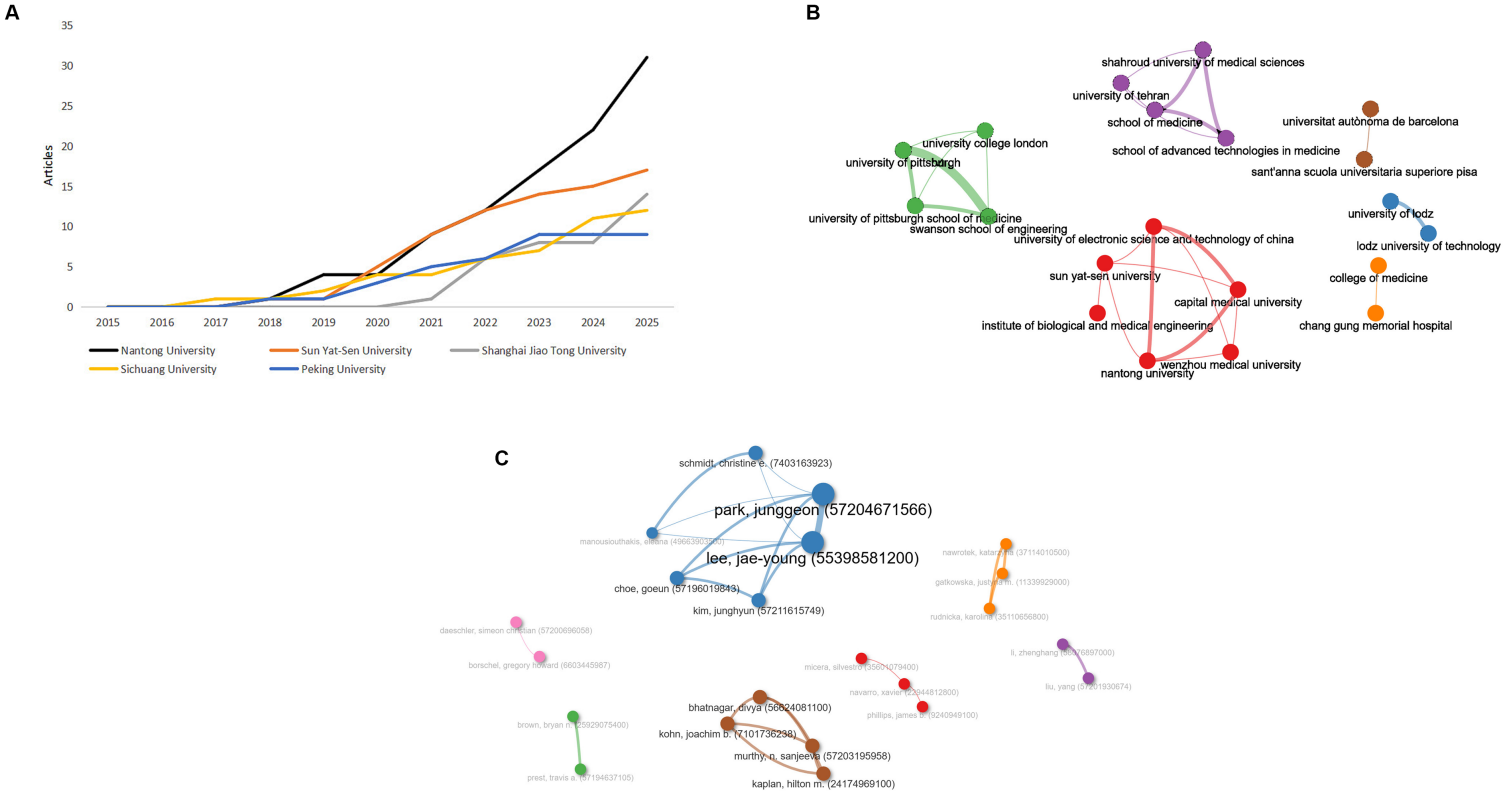
**(A)** Annual publication trends of the top five most productive institutions from 2015 to 2025; **(B)** Institutional collaboration network, where nodes represent institutions and edges indicate collaborative relationships (different colors denote distinct clusters); **(C)** Co-authorship network of core researchers, with node size proportional to publication output or impact metrics.

Temporal trends in publication output for leading Chinese institutions are detailed in Figure 5A and its accompanying data table. Nantong University exhibited the most pronounced growth, with its annual publication count escalating sharply from 4 in 2018 to 31 in 2025. Sun Yat-sen University also demonstrated a consistent and significant increase in output, reaching 17 articles by 2025. Shanghai Jiao Tong University, while starting later (first article in 2021), showed a steady rise to 14 publications by 2025. Sichuan University and Peking University also contributed to the expanding research landscape, showing incremental increases in their annual publication volumes throughout the observed period.

Co-authorship network analysis, derived from the merged international databases and visualized in Figure 5B, revealed a structure characterized by several distinct collaborative clusters. A prominent Chinese cluster (red nodes) included Nantong University, Wenzhou Medical University, Capital Medical University, Sun Yat-sen University, University of Electronic Science and Technology of China, and the Institute of Biological and Medical Engineering, indicating robust domestic collaboration. Other notable clusters encompassed a UK–US group (green nodes) featuring University College London and the University of Pittsburgh and its affiliated schools; an Iranian cluster (purple nodes) centered on Shahroud University of Medical Sciences and the University of Tehran; and a European collaboration (brown nodes) involving Universitat Autonoma de Barcelona and Sant’Anna Scuola Universitaria Superiore Pisa.

The co-authorship network among researchers in hydrogel applications for peripheral nerve repair is visualized in Figure 5C. This network revealed distinct clusters of collaboration. A prominent cluster (blue nodes) centered around Junggeon Park and Jae-young Lee, who exhibited extensive direct collaborations with researchers such as Christine E. Schmidt, Eleana Manousiouthakis, Goeun Choe, and Junghyun Kim. Another significant cluster (brown nodes) was observed, with Joachim B. Kohn as a central figure, collaborating with Divya Bhatnagar, Sanjeeva Murthy N., and Hilton M. Kaplan. Additionally, several smaller, more isolated collaborative groups were identified, including those involving Katarzyna Nawrotek (orange nodes), Zhenghang Li (purple nodes), Simeon Christian Daeschler (pink nodes), and Brown B. (green nodes).

Further bibliometric analysis of author metrics (Table 2) identified key contributors based on publication output and impact. Katarzyna Nawrotek recorded the highest number of publications (NP = 5) and an h-index of 5, along with a total citation count (TC) of 132. Tao Jie demonstrated the highest total citations (TC = 245) from 3 publications, achieving an h-index of 3. Zhang Meng contributed 4 publications with an h-index of 4. These authors began publishing in the field between 2016 and 2024, indicating active engagement in recent years.

**Table 2:** Decadal ranking of top researchers by scholarly impact metrics in Web of Science Core Collection.

Rank	Author	h_index	g_index	m_index	TC	NP	PY_start
1	Nawrotek Katarzyna	5	5	0.455	132	5	2016
2	Zhang Meng	4	4	0.667	126	4	2021
3	Tao Jie	3	3	0.3	245	3	2017
4	Xu Wanlin	3	3	0.6	61	3	2022
5	Xue Wen	3	3	0.5	190	3	2021
6	Carvalho Cristiana R.	2	2	0.222	72	2	2018
7	Dobbs Ryan	2	2	0.222	30	2	2018
8	Fang Jiaqi	2	2	0.5	10	2	2023
9	Gao Yisheng	2	2	0.667	42	2	2024
10	Huang Qun	2	2	0.333	80	2	2021

TC, Total Citations; NP, Number of Papers; PY_start, Publication Year Start.

### 3.4 Analysis of journals

As primary channels for scholarly communication, journals reflect both disciplinary focus and scientific impact within a research domain. To identify leading journals in hydrogel-based PNI research, a cross-disciplinary ranking was established by integrating data from three major bibliographic databases: WoSCC, Scopus, and PubMed. The top ten journals from each database, ranked by publication count, were initially extracted. These lists were then consolidated, and journals were re-ranked based on their cumulative publication volume across all sources. Heterogeneous and non-standardized data formats across the databases prevented unified analysis of journal collaborations and evolution. Consequently, WoSCC was selected as the sole data source due to its consistent and high-quality metadata.

Table 3 presents a consolidated ranking of leading journals publishing research on hydrogel-based peripheral nerve repair, derived from an integrated analysis of the bibliographic databases. The ranking, based on cumulative publication counts, shows that *Advanced Healthcare Materials*, *Acta Biomaterialia*, and *Bioactive Materials* are the three most prominent journals in this field. A key finding is the strong correlation between a journal’s rank and its breadth of database coverage: the top four journals are indexed in all three major databases (PubMed, Scopus, and WoSCC). In contrast, several journals, including the well-regarded *Biomaterials*, appear in the lower half of the ranking despite high publication counts in a single database, underscoring the value of a multi-database approach to mitigate indexing bias. The analysis also indicates a concentration of influential publications within a small group of publishers, notably Elsevier and Wiley, highlighting their dominant role in disseminating key research in this domain.

**Table 3:** Top 10 productive journals in hydrogel research for peripheral nerve injury across three databases (WoSCC, Scopus, and PubMed).

Rank	Journals	PubMed	Scopus	WoSCC	Total	Database coverage	Publisher
1	Advanced Healthcare Materials	6	13	10	29	3	Wiley
2	Acta Biomaterialia	7	10	11	28	3	Elsevier
3	Bioactive Materials	8	9	10	27	3	KeAi (Elsevier partnership)
4	ACS Biomaterials Science & Engineering	3	8	9	20	3	American Chemical Society
5	Journal of Biomedical Materials Research Part A	–	8	11	19	2	Wiley
6	Materials Today Bio	4	–	11	15	2	Elsevier
7	ACS Applied Materials & Interfaces	3	–	9	12	2	American Chemical Society
8	Biomaterials	–	11	–	11	1	Elsevier
9	International Journal of Molecular Sciences	–	11	–	11	1	MDPI
10	Tissue Engineering. Part A	6	–	–	6	1	Mary Ann Liebert

**Figure 6:**
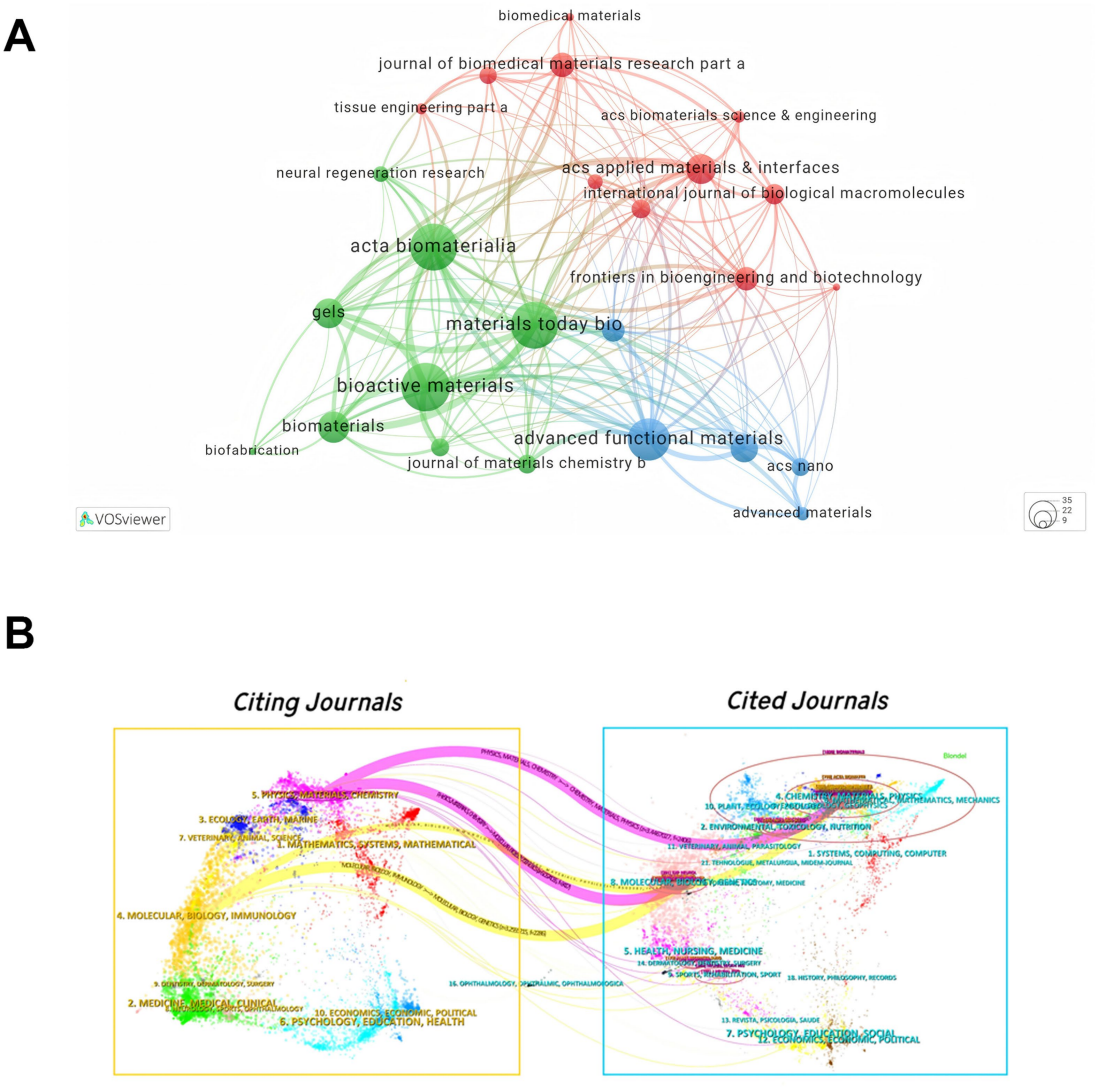
**(A)** Co-citation network of journals in hydrogel research for peripheral nerve injury. Nodes represent journals, sized according to their total link strength indicating influence. Lines between nodes show co-citation relationships. Different colors signify distinct clusters of collaboration. **(B)** The dual-map overlay of journals related to hydrogel research for peripheral nerve injury.

The VOSviewer map in Figure 6A illustrates the co-citation network of journals in the field of biomaterials and related areas. The network is divided into three distinct clusters, each represented by a different color. The green cluster, centered around *Acta Biomaterialia*, *Materials Today Bio*, and *Bioactive Materials*, focuses on biomaterials and their applications in tissue engineering and neural regeneration. The red cluster, with *ACS Applied Materials & Interfaces* as the central node, emphasizes research on biomedical materials and their interfaces. The blue cluster, anchored by *Advanced Functional Materials* and *ACS Nano*, highlights advancements in functional nanomaterials. The dual-map overlay generated by CiteSpace, comprising citing journals (left) and cited journals (right), reveals trajectories of knowledge diffusion and the evolution of research hotspots (Figure 6B). The orange and pink pathways distinctly demonstrate the disciplinary topic distribution and evolutionary patterns within the field.

### 3.5 Analysis of co-cited references

Co-citation analysis identifies intellectual connections between publications based on their joint occurrence in reference lists, thereby revealing structural shifts and emerging trends within a research field. Given the inherent citation bias associated with review articles, this analysis was restricted to Article-type publications from WoSCC.

**Figure 7:**
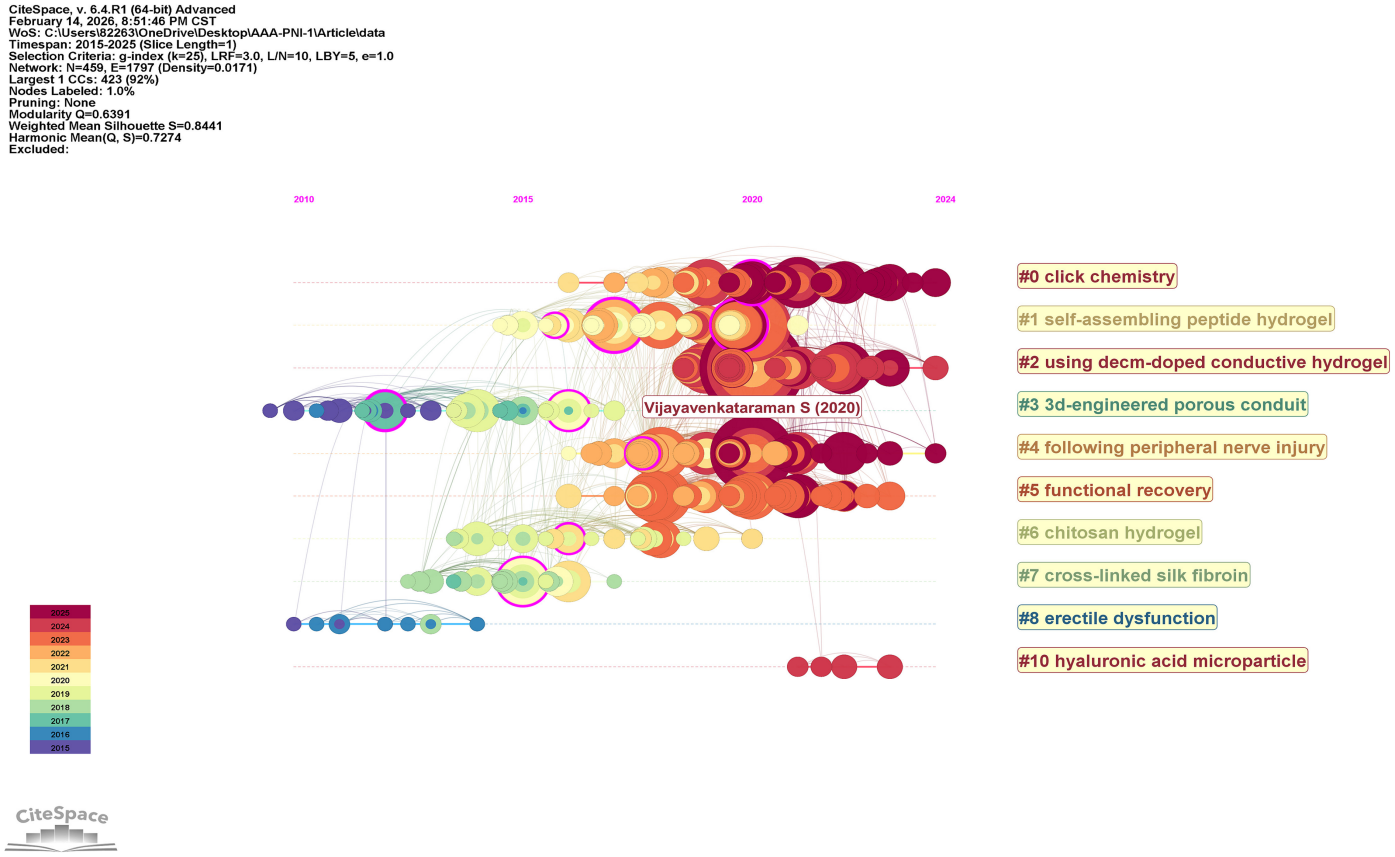
Timeline view of these co-cited reference clusters.

Figure 7 presents the timeline visualization of thematic evolution in PNI and hydrogel research from 2015 to 2025. During 2015–2020, the field established its material and methodological foundations. Seven themes emerged: material platforms including “#6 chitosan hydrogel,” “#7 cross-linked silk fibroin,” and “#1 self-assembling peptide hydrogel”; synthetic methodology through “#0 click chemistry”; functional applications via “#2 using decm-doped conductive hydrogel”; and clinical orientation through “#4 following peripheral nerve injury” and “#5 functional recovery.” Vijayavenkataraman S (2020) marked a pivotal point in this phase, synthesizing prior advances in nerve conduit design. From 2020 to 2025, the field shifted toward refinement and specialization. Four themes persisted: “#0 click chemistry,” “#2 using decm-doped conductive hydrogel,” “#4 following peripheral nerve injury,” and “#5 functional recovery,” indicating their established centrality.

The highly cited literature clusters into three functional categories in Table 4: (1) materials science and fabrication methodology (ranks 1, 3, 6, 7), encompassing systematic reviews of conduit design and innovative hydrogel synthesis (13, 14); (2) clinical and experimental therapeutics (ranks 2, 4, 5, 9, 10), spanning FDA-approved devices, autograft management, and regenerative strategies; and (3) fundamental biological mechanisms (rank 8), specifically macrophage-mediated vascularization in nerve repair. The temporal distribution of these citations—with 50% of top-cited articles published between 2015 and 2020—underscores the rapid knowledge turnover characteristic of this field. The review by Vijayavenkataraman S (2020), published in *Acta Biomaterialia* and titled “Nerve guide conduits for peripheral nerve injury repair: A review on design, materials and fabrication methods,” is widely regarded as a foundational work in the field of peripheral nerve injury repair (15). Its significance is underscored by 50 citations and its top position (No. 1) in Table 4. The article provided a comprehensive overview of nerve guide conduit approaches from 2015 to 2020. It systematically detailed various design strategies, including hollow, porous, grooved, multi-channel, and fiber-filled configurations. Furthermore, it explored the diverse material systems employed, encompassing both natural and synthetic polymers, alongside a review of fabrication methods, from traditional casting to advanced 3D printing techniques. Looking ahead, the review presciently highlighted cell-seeded conduits, growth factor delivery, and 4D printing as promising avenues for future research and development.

**Table 4:** Top 10 most cited publications in peripheral nerve injury and hydrogel in WoSCC (2015–2025).

Rank	Cited references	Citations	Journal	Author	Year
1	Nerve guide conduits for peripheral nerve injury repair: A review on design, materials and fabrication methods	(50)	Acta Biomaterialia	Sanjairaj Vijayavenkataraman	2020
2	Neural tissue engineering options for peripheral nerve regeneration	(37)	Biomaterials	Xiaosong Gu	2014
3	Functional evaluation of complete sciatic, peroneal, and posterior tibial nerve lesions in the rat	(35)	Plastic and Reconstructive Surgery	J R Bain	1989
4	Peripheral nerve reconstruction after injury: a review of clinical and experimental therapies	(34)	Biomed Research International	D Grinsell	2014
5	FDA approved guidance conduits and wraps for peripheral nerve injury: a review of materials and efficacy	(33)	Injury	S Kehoe	2012
6	Electrically Conductive Hydrogel Nerve Guidance Conduits for Peripheral Nerve Regeneration	(32)	Advanced Functional Materials	Junggeon Park	2020
7	Aligned chitosan nanofiber hydrogel grafted with peptides mimicking bioactive brain-derived neurotrophic factor and vascular endothelial growth factor repair long-distance sciatic nerve defects in rats	(31)	Theranostics	Feng Rao	2020
8	Macrophage-Induced Blood Vessels Guide Schwann Cell-Mediated Regeneration of Peripheral Nerves	(29)	Cell	Anne-Laure Cattin	2015
9	Peripheral nerve regeneration: experimental strategies and future perspectives	(28)	Advanced Drug Delivery Reviews	Alessandro Faroni	2015
10	Management of nerve gaps: autografts, allografts, nerve transfers, and end-to-side neurorrhaphy	(25)	Experimental Neurology	Wilson Z. Ray	2010

### 3.6 Analysis of co-occurring keywords and burst terms

Keyword analysis is a core bibliometric method for mapping knowledge domains tracking research evolution and detecting emerging trends. To identify novel research frontiers we conducted comparative analyses between two datasets: The single WoSCC database and the deduplicated database after merging WoSCC PubMed and Scopus. Only original research articles were included; review articles were excluded to ensure focus on primary research findings.

**Figure 8:**
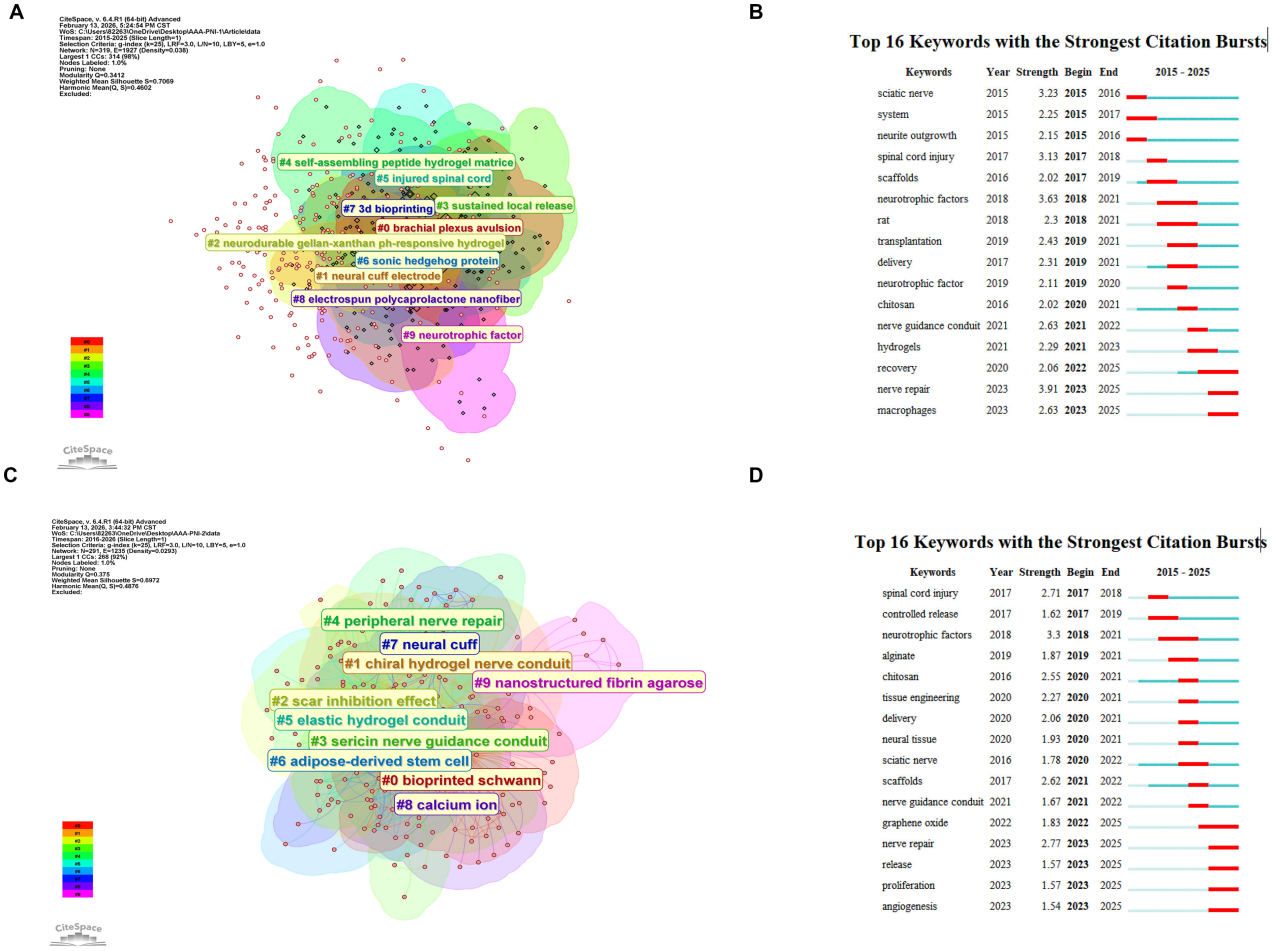
Keyword analysis of literature from WoSCC and the deduplicated database after merging WoSCC, PubMed, and Scopus. **(A)** Clustering analysis of keyword co-occurrence networks from WoSCC. **(B)** Top 16 keywords with the strongest citation bursts based on WoSCC. **(C)** Clustering analysis of keyword co-occurrence networks from the deduplicated database. **(D)** Top 16 keywords with the strongest citation bursts based on the deduplicated database.

Figure 8A presents a cluster view of the knowledge network derived from WoSCC. The largest clusters reveal foundational and emerging themes: self-assembling peptide hydrogel matrices, injured spinal cord, and sonic hedgehog protein highlight the early focus on the biological microenvironment, while sustained local release and 3D bioprinting signify a shift toward advanced manufacturing and drug delivery systems. The presence of brachial plexus avulsion indicates attention to specific clinical injuries. Figure 8B shows citation bursts over time: from 2015 to 2017, terms like sciatic nerve and neurite outgrowth established basic research models; from 2018 to 2021, neurotrophic factors and delivery surged, reflecting interest in biochemical agents; from 2022 to 2025, nerve repair became central, with macrophages and recovery highlighting growing attention to immune response and functional outcomes.

Figure 8C represents a merged dataset from WoSCC, PubMed, and Scopus. Clusters such as chiral hydrogel nerve conduit, sericin nerve guidance conduit, and elastic hydrogel conduit dominate, indicating a shift toward engineering optimal conduits. The presence of adipose-derived stem cells and bioprinted Schwann cells reflects integration of cell therapy with biomaterials, while scar inhibition effect points to strategies that actively inhibit regenerative barriers. Figure 8D reveals citation bursts in the merged dataset: controlled release, neurotrophic factors, and chitosan confirm their fundamental importance across databases. A notable recent burst is graphene oxide from 2022 to 2025, highlighting an emerging material trend. The latest bursts from 2023 to 2025 include nerve repair, proliferation, angiogenesis, and release, representing a holistic view that combines neural regeneration, vascularization, and controlled delivery.

Comparing the single-database (WoSCC) and multi-database analyses offers crucial insights into hydrogel research for nerve repair. Both agree on the indispensable roles of neurotrophic factors and controlled delivery systems. While WoSCC broadly highlighted “hydrogels,” the combined dataset provided greater granularity, identifying chiral, elastic, and sericin-based conduits as specific hotspots—reflecting a field increasingly focused on precise material properties. The multi-database analysis uniquely revealed graphene oxide and angiogenesis as significant recent emerging themes, bridging materials science and vascular biology, likely underrepresented in WoSCC alone. This, coupled with WoSCC’s late burst in “macrophages” and the merged data’s emphasis on “angiogenesis” and “proliferation,” signifies a clear evolution: from merely providing a scaffold to actively engineering a comprehensive regenerative microenvironment that modulates immune response, promotes vascularization, and supports neural growth. Notably, burst analysis identifies “spinal cord injury” as an emerging research frontier (2017–2018), signaling a translational expansion of hydrogel technologies from peripheral to central nervous system applications. Building upon earlier foundational work in this field, the fibrin-based “reviving matrix” system was initially validated in rabbit chronic peripheral nerve injury models (16) and subsequently applied to rat models of complete spinal cord injury (17), demonstrating cross-system therapeutic potential. This extension from PNI to central nervous system repair suggests that future hydrogel designs must account for broader neural regeneration microenvironments beyond peripheral contexts. In essence, the odyssey of hydrogels in nerve repair is now firmly in a sophisticated phase, centered on intelligent design to direct complex biological processes for true functional recovery.

## 4 Discussion

### 4.1 Global research dynamics: publication trends and collaborative landscape

#### 4.1.1 Publication trends and field maturation

Publication numbers across major databases demonstrated a consistent upward trajectory from 2015 to 2023, followed by a slight moderation in 2024. This pattern suggests a potential inflection point in the field’s development. While partly attributable to research and publication disruptions stemming from the global COVID-19 pandemic, this moderation could concurrently signal a shift from rapid expansion to a more mature phase—a hypothesis further supported by our broader bibliometric findings. The trajectory in publication numbers across major databases following the 2023 peak suggests a potential inflection point in the field’s development. This decline may signal a transition from rapid expansion to a maturation phase, a hypothesis supported by several observations from our bibliometric data (18, 19).

#### 4.1.2 Global research landscape and collaborative networks

As the field matures, bibliometric analysis reveals a concentrated yet collaborative global research landscape in hydrogel applications for peripheral nerve repair. China unequivocally leads in research output with 248 documents (55.2% of the top 10 countries), though its average citations per article (21.69) trails behind Canada’s exceptional 57.56, indicating potential for enhanced research impact. The United States maintains a solid second position with 90 documents and 1,398 total citations, demonstrating consistent quality alongside quantity.

International collaboration forms distinct geographic patterns: Cluster 1 (green) encompasses Iran, Italy, and Switzerland; Cluster 2 (blue) groups Germany, Spain, and the United Kingdom; Cluster 3 (orange) comprises Canada, Japan, and the United States; Cluster 4 (purple) connects India and South Korea; while China alone constitutes Cluster 5 (red), reflecting its dominant yet somewhat independent research ecosystem.

The temporal analysis (Figure 4B) vividly illustrates China’s explosive growth trajectory, particularly post-2020, which has surged far beyond all other nations. In contrast, the United States demonstrates steady, linear growth, maintaining consistent contribution throughout the decade. This divergence suggests different research development models—China’s rapid scaling versus the United States’ sustained incremental advancement.

At the institutional level, Nantong University exhibited the most pronounced growth, escalating from 4 publications in 2018 to 31 in 2025. Sun Yat-sen University and Shanghai Jiao Tong University also demonstrated consistent increases, reaching 17 and 14 articles, respectively, by 2025. The co-authorship network reveals robust domestic collaboration within China, alongside established transatlantic partnerships (University College London—University of Pittsburgh) and emerging regional clusters (Iranian and European networks).

### 4.2 Hotspots and future research directions

#### 4.2.1 Empirical evidence from bibliometrics on research focus and emerging trends

Keyword clustering and burst detection revealed four prominent and interconnected themes within the hydrogel-based peripheral nerve injury literature: angiogenesis conductive hydrogels 3D bioprinting and nerve guidance conduits. These themes collectively reflect the field’s evolution toward multifunctional biomaterials advanced fabrication techniques and a more comprehensive understanding of the regenerative microenvironment.

Angiogenesis has emerged as a critical consideration in recent years, reflecting the growing recognition that neural regeneration cannot occur in isolation from vascular support. Figure 8D provides the clearest evidence of this trend, with “angiogenesis” appearing as a burst keyword from 2023 to 2025 (strength 1.54). This recent surge indicates that researchers are increasingly focused on engineering constructs that promote blood vessel formation alongside axon guidance (20). The temporal alignment of angiogenesis with other 2023–2025 bursts—including “nerve repair” (strength 2.77), “proliferation” (strength 1.57), and “release” (strength 1.57)—suggests a holistic approach to tissue engineering that simultaneously addresses neural regeneration, vascularization, and controlled bioactive factor delivery (21). The inclusion of vascular considerations represents a paradigm shift from viewing nerve guidance conduits as passive physical scaffolds to recognizing them as active participants in orchestrating complex multicellular processes.

Conductive Hydrogels emerged as a distinct research focus aimed at providing electrical cues to enhance neurite outgrowth and functional recovery. Figure 7 features cluster #2 explicitly labeled “using decom-doped conductive hydrogel,” referring to conductive polymer-doped systems that combine hydrogel flexibility with electrical conductivity. The importance of conductive properties is further underscored by the strong recent burst of “graphene oxide” (strength 1.83, 2022–2025) in Figure 8D, indicating substantial interest in incorporating conductive nanomaterials into hydrogel matrices (22). Additionally, Figure 8A includes cluster #2 “neurodurable gellan-xanthan pH-responsive hydrogel,” which, while not explicitly conductive, suggests a parallel trend toward stimuli-responsive materials that may eventually integrate conductive components (23). The convergence of conductive materials with traditional hydrogels aims to mimic the native electrical environment of neural tissue and provide contact guidance cues that direct axon extension.

3D Bioprinting represents a transformative manufacturing paradigm that enables precise spatial control over scaffold architecture and cellular distribution (24, 25). Figure 8A features cluster #7 “3d bioprinting,” establishing this technique as a major thematic area within the single-database analysis. Figure 8C further refines this theme with cluster #0 “bioprinted Schwann,” indicating the integration of living Schwann cells into printable bioinks for creating cellularized constructs that more closely mimic native nerve structure (26). Figure 7 contributes cluster #3 “3d-engineered porous conduit,” emphasizing the application of printing technologies to fabricate conduits with patient-specific geometries and precisely controlled porosity. Temporal burst analysis reveals the maturation of this field: Figure 8B shows “scaffolds” (strength 2.02, 2017–2019) and “nerve guidance conduit” (strength 2.63, 2021–2022) were active during periods when bioprinting techniques were being optimized, while Figure 8D confirms sustained interest in “scaffolds” (strength 2.62, 2021–2022) and “nerve guidance conduit” (strength 1.67, 2021–2022). The appearance of bioink-relevant materials such as “alginate” (strength 1.87, 2019–2021) and “chitosan” (strength 2.55, 2020–2021) in Figure 8D further supports the development of printable hydrogel formulations with appropriate rheological properties for extrusion-based printing (21, 26, 27).

Nerve Guidance—the overarching goal of engineered conduits—permeates multiple clusters and bursts across all figures. Figure 8C is particularly rich in conduit-focused clusters: #1 “chiral hydrogel nerve conduit,” #3 “sericin nerve guidance conduit,” and #5 “elastic hydrogel conduit,” each representing different material strategies for guiding regenerating axons through physical, chemical, or topographical cues. Figure 8A contributes cluster #8 “electrospun polycaprolactone nanofiber,” a common conduit fabrication method that produces aligned fibers to direct axon growth, and cluster #1 “neural cuff electrode,” which interfaces with nerves for electrical stimulation (28). Burst detection in both datasets consistently highlights “nerve guidance conduit” as a key term (Figure 8B: 2021–2022; Figure 8D: 2021–2022). The recent bursts of “nerve repair” (Figure 8B: strength 3.91, 2023–2025; Figure 8D: strength 2.77, 2023–2025) and “recovery” (Figure 8B: 2022–2025) signal a translational shift from materials characterization toward functional outcomes assessment (29). The simultaneous emergence of “angiogenesis” (Figure 8D: 2023–2025) and “macrophages” (Figure 8B: 2023–2025) alongside nerve guidance themes indicates that successful regeneration strategies must now address the vascular and immune microenvironments as integrated components of conduit design. Collectively, these four themes illustrate a multidisciplinary evolution where conductive functionality, advanced bioprinting techniques, optimized conduit architectures, and pro-angiogenic strategies converge to enhance peripheral nerve regeneration through increasingly sophisticated and biologically informed approaches.

#### 4.2.2 Pathological alterations following PNI: the emerging importance of angiogenesis in regenerative strategies

The pathological alterations following peripheral nerve injury are complex and dynamic, representing a critical barrier to effective regeneration while providing clear targets for the design of biomaterials such as hydrogels (30). Upon injury, distal axons undergo rapid Wallerian degeneration, characterized by axonal fragmentation and subsequent clearance to establish a permissive environment for regrowth. In contrast, proximal axons attempt to form regenerative sprouts; however, their elongation is often hindered by inadequate guidance and insufficient trophic support (31). Concurrently, the local microvascular network is severely disrupted, leading to immediate hypoxia and nutrient deprivation that exacerbates neuronal death and delays the regenerative response (32). In addition to this, a robust local inflammatory response is activated, marked by elevated levels of pro-inflammatory cytokines (e.g., IL-1β, IL-6) and a reduction in anti-inflammatory cytokines (e.g., IL-10). This imbalanced microenvironment further impedes successful nerve regeneration (33).

The phenotypic switch of Schwann cells (SCs) is a central event in the repair process. Upon injury, SCs dedifferentiate into a repair phenotype, proliferate, and align into Büngner bands in an attempt to guide axonal regrowth and initiate remyelination (33). Notably, the formation of these regenerative corridors is critically dependent on concomitant angiogenesis, as newly formed blood vessels provide both physical scaffolds for axonal guidance and essential trophic support including oxygen, nutrients, and endothelial cell-derived neurotrophic factors (34). Specifically, endothelial cell-secreted exosomes have been demonstrated to actively promote Schwann cell repair phenotype transition, enhancing their proliferation, migration, and secretion of pro-regenerative factors (35, 36). However, this process is highly susceptible to dysregulation. Studies have shown that the absence of molecular chaperones such as αB-crystallin impairs remyelination, leading to a reduction in myelinated SCs and disruption of critical signaling interactions with axons—such as the neuregulin/ErbB2 pathway—ultimately compromising functional recovery (37–39).

More severely, injury can trigger abnormal changes in the somata of sensory neurons and even induce “transganglionic degeneration” of their central terminals, resulting in deafferentation of the dorsal horn. This may serve as a structural basis for neuropathic pain and maladaptive reorganization of central circuitry (40–42). The chronic ischemia resulting from failed angiogenesis further exacerbates this pathological cascade by promoting oxidative stress and sustained neuroinflammation, creating a self-perpetuating cycle of degeneration (32).

Conventional repair strategies, such as direct suture or autologous nerve grafting, exhibit limited efficacy in addressing long-segment nerve defects. This limitation is particularly pronounced in the context of vascular compromise, as autografts often suffer from inadequate neovascularization at the host-graft interface, leading to central necrosis and poor functional integration (43). Consequently, tissue engineering approaches, particularly the development of pro-angiogenic hydrogel-based nerve guidance conduits, have emerged as a prominent research focus (44). Incorporating pro-angiogenic properties into these conduits has become an attractive strategy to enhance vascularization and, in turn, improve regenerative outcomes. These advanced biomaterials are specifically designed to actively promote angiogenesis through sustained delivery of angiogenic factors such as vascular endothelial growth factor (VEGF), incorporation of endothelial cell-friendly extracellular matrix components, or biomimetic topographical cues that guide vascular ingrowth alongside neural regeneration (20–22). The strategic integration of pro-angiogenic functionality represents a paradigm shift from passive structural support to active microenvironmental modulation, recognizing that robust vascularization is not merely beneficial but absolutely essential for successful nerve repair across clinically relevant defect lengths (32).

#### 4.2.3 “Dynamic electro-chemical coupling” in conductive hydrogels

Building upon the understanding of PNI pathophysiology, bibliometric analysis indicates that conductive hydrogels remain a prominent research focus. However, the majority of current studies remain confined to examining the correlation between static electrical conductivity and axonal extension, overlooking the critical time-dependent attenuation of the electrophysiological microenvironment during nerve regeneration (45). In terms of material development, conductive hydrogels have evolved from early strategies reliant on simple physical blending of conductive fillers with inert matrices toward integrated functional systems that synergistically combine intrinsically conductive polymers with dynamic covalent/non-covalent crosslinked networks (45).

To address this challenge, future efforts should prioritize the development of intelligent conductive hydrogel systems with programmable electrical conductivity. One promising approach involves the incorporation of cleavable oligoaniline sequences or reactive oxygen species (ROS)-responsive pyrrole motifs, enabling conductivity to adaptively decrease in accordance with the resolution of inflammation—thus dynamically aligning with the evolving demands of nerve regeneration (46, 47). Alternatively, integrating piezoelectric or triboelectric nanodomains into the hydrogel network offers a pathway to harness physiological mechanical stimuli, such as muscle contractions, for *in situ* electrical pulse generation without external power sources (46, 48, 49). This strategy facilitates the creation of a self-powered electroactive microenvironment that is intrinsically coupled to biological activity, thereby obviating the need for secondary surgeries to remove implanted electrodes and mitigating associated clinical complications (50).

Such innovative strategies, which emphasize dynamic regulation and bioenergetic autonomy, are expected to significantly enhance the sustained efficacy of electrical stimulation in long-distance nerve regeneration. This approach addresses a critical limitation of traditional static electroactive scaffolds, whose stimulatory functionality gradually declines during long-term implantation (51).

#### 4.2.4 3D/4D bioprinting: precision manufacturing and dynamic adaptability

In parallel to advancing material chemistry, the field is being transformed by breakthroughs in fabrication technology. 3D and 4D bioprinting represent transformative approaches for fabricating advanced nerve guidance conduits (NGCs). Unlike traditional methods, 3D bioprinting enables precise fabrication of scaffolds with customized architectures, such as multi-luminal channels and neurotrophic factor gradients, which provide topographical and biochemical cues for directed axonal growth and Schwann cell migration (52, 53).

4D bioprinting extends this capability by employing stimuli-responsive bioinks that enable dynamic morphological changes post-implantation. For instance, temperature-triggered shape transformation allows deployment of minimally invasive conduits that self-expand to bridge nerve defects (54, 55). This enhances surgical handling and improves anatomical integration.

Current challenges include balancing bioink printability with biocompatibility and achieving vascularization within thick constructs (56, 57). Future directions should focus on developing multi-material bioinks that combine printability, conductivity, and bioactivity. The integration of 4D printing with self-healing materials could yield next-generation implants that maintain structural integrity throughout regeneration (51, 55).

#### 4.2.5 Advancing nerve guidance conduits through hydrogel functionalization

The convergence of functional hydrogels and advanced manufacturing naturally leads to the evolution of nerve guidance conduit (NGC) itself. The combination of these two components creates a synergistic system that overcomes their individual limitations. Hydrogel-filled NGCs transform passive conduits into bioactive regenerative platforms by providing internal microarchitectural guidance and sustained molecular release (58). This integration enables three key advantages: First, the conduit maintains structural integrity during implantation and provides directional guidance, while the internal hydrogel creates a biomimetic microenvironment that promotes cellular activities (59). Second, hydrogels serve as excellent carriers for controlled delivery of neurotrophic factors (e.g., Nerve Growth Factor (NGF), Glial cell line-Derived Neurotrophic Factor (GDNF)) and extracellular matrix components, addressing the biological inertia of pure synthetic conduits (53). Third, advanced manufacturing techniques such as 3D printing allow creation of multi-luminal conduits with spatially organized hydrogel fillers that can simultaneously support sensory and motor axon regeneration through differential biofactor release (7, 58).

This integrated strategy marks a significant shift in nerve repair, transitioning from passive tubular bridging to actively guided regeneration (59). Future research should prioritize optimizing conduit–hydrogel interfaces and developing intelligent, responsive systems capable of adapting to physiological stimuli (58).

### 4.3 Limitations

This study has several constraints. First, our analysis was limited to English-language publications, potentially omitting relevant non-English studies. Second, the October 2025 cutoff likely underrepresents recent advances, particularly 2023–2025 burst keywords (e.g., “angiogenesis,” “macrophages”), given indexing delays. Third, quantitative metrics cannot assess methodological rigor or translational impact—high Chinese publication volume (55.2%) does not equate to proportional clinical implementation. Notably, despite intense research activity, clinical translation remains severely limited: Food and Drug Administration (FDA)-approved hydrogel-based nerve guidance conduits are virtually absent, with current clinical options restricted to acellular synthetic conduits that lack the bioactive, cell-modified, and stimuli-responsive features driving academic research. This disconnect between bibliometric hotspots and regulatory approval highlights the need for standardized manufacturing protocols, long-term safety data, and proactive regulatory science engagement. Fourth, excluding reviews from co-citation analyses may have filtered influential syntheses. These limitations do not invalidate the core findings but underscore the urgency of bridging the laboratory-clinic gap.

## 5 Conclusion

This bibliometric analysis reveals that hydrogel-based peripheral nerve repair has evolved from passive scaffolding to active microenvironmental orchestration. Four interconnected pillars now define the field: conductive hydrogels, 3D/4D bioprinting, pro-angiogenic engineering, and immunomodulatory strategies. The 2023–2025 emergence of “angiogenesis,” “nerve repair,” and “macrophages” as strongest bursts signals a paradigm shift toward biologically informed design.

However, critical gaps persist: standardized bioinks, long-term *in vivo* validation, integration of pro-angiogenic and immunomodulatory approaches, and regulatory pathways for multifunctional conduits. Future priorities should focus on stimuli-responsive “smart” hydrogels, interdisciplinary collaboration, large animal preclinical validation, and clinical translation infrastructure to accelerate progress from research hotspots to patient outcomes.

## Data Availability

The original contributions presented in the study are included in the article/Supplementary material, further inquiries can be directed to the corresponding author.
