# Supplementary material for: Hydrogel research in peripheral nerve injury repair: a comprehensive multi-database bibliometric analysis (2015–2025)
Source: Front Neurol. 2026 Apr 29;17:1763950. doi: 10.3389/fneur.2026.1763950 (PMC13167558; doi:10.3389/fneur.2026.1763950)
Supplement: Supplementary file 1 [file Supplementary_file_1.docx]

**Hydrogel Research in Peripheral Nerve Injury Repair: A Comprehensive Multi Database Bibliometric Analysis Revised Manuscript with Detailed Methodology-**

**Detailed Methodology**

1. **Materials and Methods**

**1.1 Search Strategy**

**1.1.1 Web of Science Core Collection (WoSCC) Search**

Search String:

TS=(("peripheral nerve injury" OR "peripheral nerve damage" OR

"traumatic peripheral nerve injury" OR "peripheral nerve regeneration" OR "sciatic nerve injury" OR "nerve gap")

AND

("hydrogel" OR "hydrogel scaffold" OR "hydrogel conduit"))

Search Parameters:

Database: Web of Science Core Collection

Time span: 2015-01-01 to 2025-10-01

Document types: Article, Review

Languages: English

Search date: October 1, 2025

Results: 374 records

**1.1.2 Scopus Search**

Search String:

(TITLE-ABS-KEY(("peripheral nerve injury" OR "peripheral nerve damage" OR

"traumatic peripheral nerve injury" OR "peripheral nerve regeneration" OR "sciatic nerve injury" OR "nerve gap")

AND

("hydrogel" OR "hydrogel scaffold" OR "hydrogel conduit")))

Search Parameters:

Database: Scopus (Elsevier)

Search fields: Title, Abstract, Keywords (TITLE-ABS-KEY)

Date range: 2015 to 2025

Document types: Article, Review

Language: English

Search date: October 1, 2025

Results: 287 records

**1.1.3 PubMed Search**

Search String:

(Title/Abstract(("peripheral nerve injury" OR "peripheral nerve damage" OR

"traumatic peripheral nerve injury" OR "peripheral nerve regeneration" OR "sciatic nerve injury" OR "nerve gap")

AND

("hydrogel" OR "hydrogel scaffold" OR "hydrogel conduit")))

Search Parameters:

Database: PubMed/MEDLINE (NCBI)

Search fields: MeSH Terms, Title/Abstract

Publication date: 2015/01/01 to 2025/10/01

Article types: Journal Article, Review

Language: English

Species: Humans or Other Animals (excluded in vitro only)

Search date: October 1, 2025

Results: 135 records

**1.2 Study Selection and Data Preprocessing**

**1.2.1 Deduplication Workflow**

Records from all three databases were merged and deduplicated using a hierarchical, rule-based approach. The deduplication process was conducted in two stages: (1) automatic deduplication using DOI matching, followed by (2) manual verification of potential duplicates. Deduplication Hierarchy and Matching Keys.

Primary Key (Level 1): DOI (Digital Object Identifier)

Exact match on normalized DOI (lowercase, stripped of whitespace)

Priority for retention: WoSCC > Scopus > PubMed

Rationale: DOI provides the most reliable unique identifier

Secondary Key (Level 2): Exact Title Match

Applied to records without DOI

Case-insensitive comparison after normalization

Punctuation and extra whitespace removed

Tertiary Key (Level 3): Title + Year + First Author

Applied when titles are similar but not exact

All three components must match

Used for detecting variants (e.g., early online vs. final version)

Software and Settings: Deduplication was performed using custom Python scripts (pandas library) with the following workflow:

Deduplication Algorithm:

1. Import records from all three databases

2. Standardize field names and formats

3. Normalize DOI: lowercase, strip whitespace

4. Group records by normalized DOI

5. For each duplicate group:

- Retain record from highest-priority database (WoSCC > Scopus > PubMed)

- Merge metadata from all versions (keywords, citations)

1. Export deduplicated dataset for screening

**1.2.2 Deduplication Results**


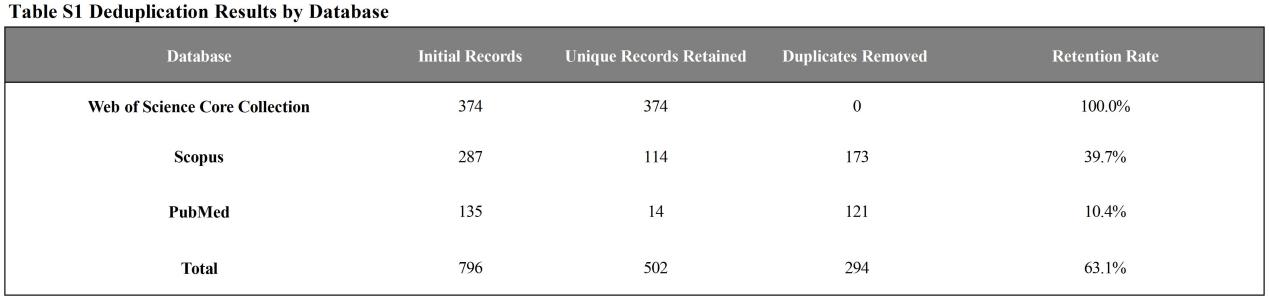


Overlap Analysis: The high deduplication rate (37% of records removed) reflects substantial overlap between databases. WoSCC contributed the most unique records (74.5% of final dataset), followed by Scopus (22.7%) and PubMed (2.8%). This pattern is consistent with the broader coverage of WoSCC in materials science and biomedical engineering.

**1.2.3 Eligibility Screening**

After deduplication, 502 unique records underwent title and abstract screening against predefined inclusion criteria:

**Inclusion Criteria:**

Studies addressing hydrogels in peripheral nerve injury/repair

Original research articles or review articles

Published between January 1, 2015 and October 1, 2025

English language

**Exclusion Criteria:**

Conference abstracts, book chapters, editorials, commentaries

Studies focusing solely on central nervous system (CNS) injury

In vitro studies without in vivo validation

Non-English publications

Two reviewers independently screened titles and abstracts; conflicts were resolved by consensus or consultation with a third reviewer. After screening, 502 articles were retained for bibliometric analysis.

**1.3 Bibliometric Analysis**

**
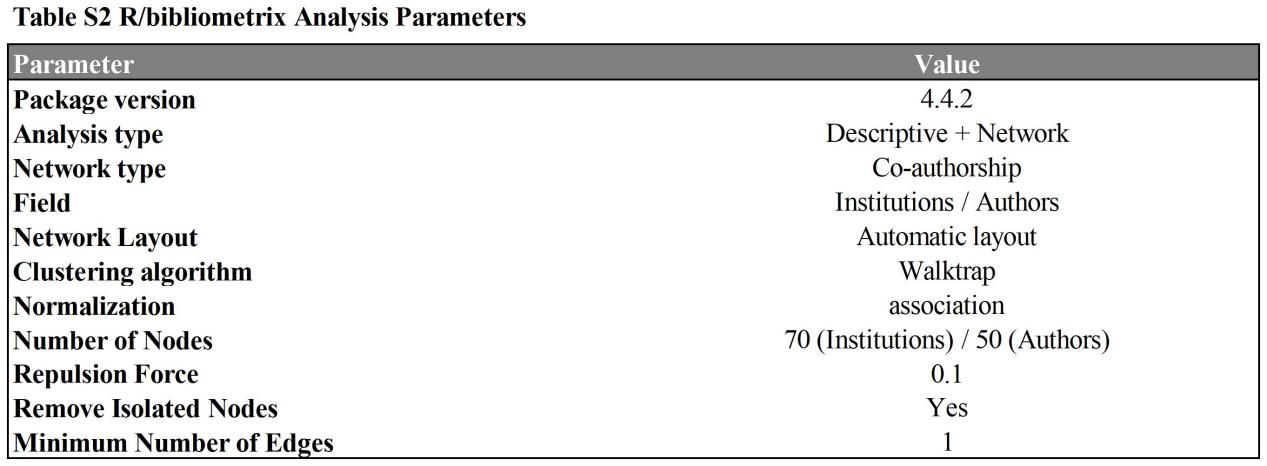
**

**
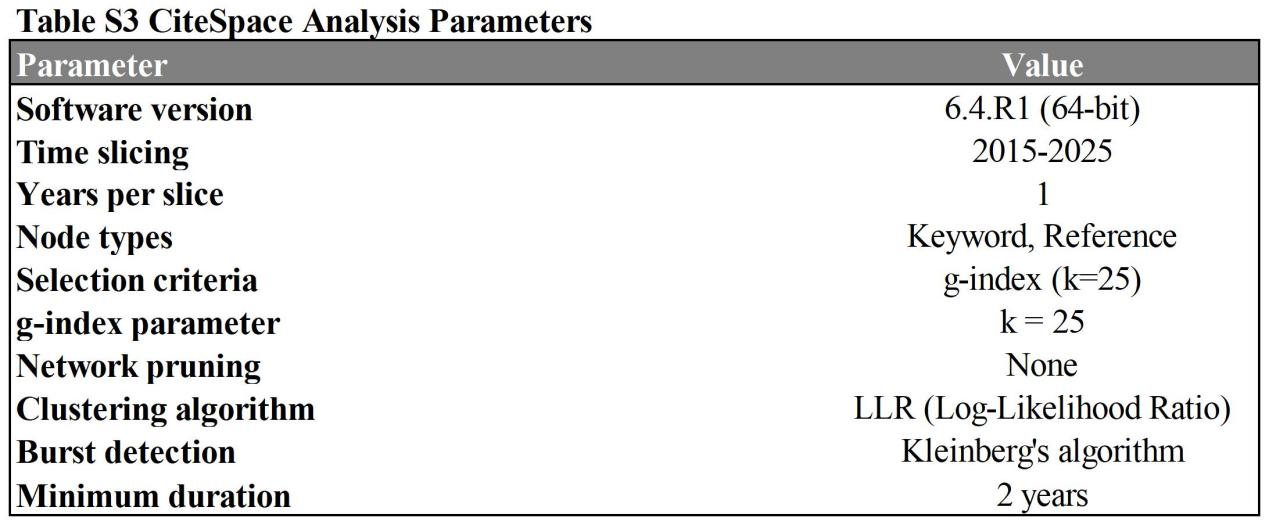
**

**
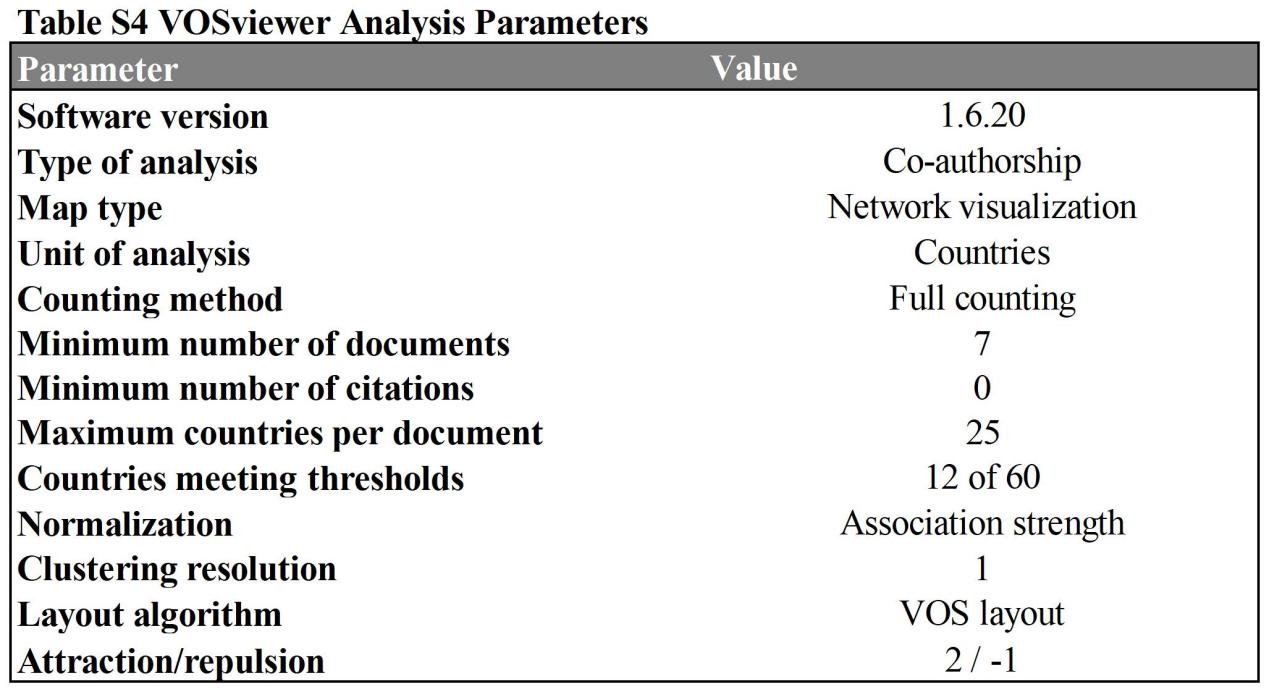
**
